# The Role of Auditory Feedback at Vocalization Onset and Mid-Utterance

**DOI:** 10.3389/fpsyg.2018.02019

**Published:** 2018-10-25

**Authors:** Nichole E. Scheerer, Jeffery A. Jones

**Affiliations:** ^1^Department of Psychology, Simon Fraser University, Burnaby, BC, Canada; ^2^Department of Psychology, Laurier Centre for Cognitive Neuroscience, Wilfrid Laurier University, Waterloo, ON, Canada

**Keywords:** auditory feedback, speech motor control, fundamental frequency, vocal pitch, event-related potential (ERP)

## Abstract

Auditory feedback plays an important role in monitoring and correcting for errors during speech production. Previous research suggests that at vocalization onset, auditory feedback is compared to a sensory prediction generated by the motor system to ensure the desired fundamental frequency (F0) is produced. After vocalization onset, auditory feedback is compared to the most recently perceived F0 in order to stabilize the vocalization. This study aimed to further investigate whether after vocalization onset, auditory feedback is used strictly to stabilize speakers’ F0, or if it is also influenced by the sensory prediction generated by the motor system. Event-related potentials (ERP) were recorded while participants produced vocalizations and heard the F0 of their auditory feedback perturbed suddenly mid-utterance by half a semitone. For half of the vocalizations, at vocalization onset, participants’ F0 was also raised by half a semitone. Thus, half of the perturbations occurred while participants heard their unaltered auditory feedback, and the other half occurred in auditory feedback that had also been perturbed 50 cents at vocalization onset. If after vocalization onset auditory feedback is strictly used to stabilize speakers’ F0, then similarly sized vocal and ERP responses would be expected across all trials, regardless of whether the perturbation occurred while listening to altered or unaltered auditory feedback. Results indicate that the perturbations to the participants’ unaltered auditory feedback resulted in larger vocal and N1 and P2 ERP responses than perturbations to their altered auditory feedback. These results suggest that after vocalization onset auditory feedback is not strictly used to stabilize speakers’ F0, but is also used to ensure the desired F0 is produced.

## Introduction

Speech is arguably the most important form of human communication (Hutchins and Moreno, 2013). Since the goal of speech production is the transfer of information, speech production must be carefully regulated to ensure the desired information is conveyed. During speech production sensory feedback, such as auditory feedback, plays an important role in maintaining the fluidity of speech, as it allows speech motor movements to be monitored and production errors to be detected and corrected (Guenther, 2006). The importance of auditory feedback during the acquisition of speech is demonstrated by individuals that are born deaf, who often fail to acquire fluent speech (Svirsky et al., 2004). Similarly, the role of auditory feedback in regulating and maintaining fluent speech is highlighted by observing individuals who become deaf post-lingually; they typically experience a gradual deterioration of the quality of their speech (Cowie et al., 1982; Goehl and Kaufman, 1984). Despite the obvious importance of auditory feedback for speech motor control, there are many unknowns with regards to how auditory feedback is utilized to regulate speech in different contexts.

The role of auditory feedback for speech motor control is often investigated by exposing participants to frequency altered feedback (FAF), auditory feedback that has been synthetically altered so that the fundamental frequency (F0) the speaker hears is either higher or lower than what the speaker is producing. Exposing speakers to FAF by inducing brief unpredictable perturbations to the F0 of their auditory feedback (Burnett et al., 1997, 1998; Burnett and Larson, 2002; Jones and Munhall, 2005; Liu et al., 2010a,b; Scheerer et al., 2013a,b; Scheerer and Jones, 2014; Tumber et al., 2014; Behroozmand et al., 2015; Scheerer, 2016; Scheerer and Jones, 2017), has consistently been shown to elicit a rapid compensatory response. This compensatory response, which usually occurs with a latency of approximately 100–150 ms (Burnett et al., 1997, 1998), has been termed the “pitch-shift reflex” (Burnett et al., 1998; Burnett and Larson, 2002; Bauer and Larson, 2003; Bauer et al., 2006; Liu et al., 2010a, 2011). Due to its reflexive nature, this response has been suggested to play a role in stabilizing voice F0 around a desired target (Hain et al., 2000; Natke et al., 2003; Bauer et al., 2006; Hawco and Jones, 2009). That being said, it is unclear whether this F0 target is fixed, as would be the case if the speaker was comparing their auditory feedback with a sensorimotor representation of the intended F0, or variable, as would be the case if the speaker was comparing their auditory feedback with the auditory feedback experienced prior to the F0 perturbation.

Larson et al. (2001) examined this question by exposing speakers to FAF that either altered the F0 of the speaker’s auditory feedback mid-vocalization (the “on” condition), or altered the F0 of the speaker’s auditory feedback prior to vocalization onset, and then removed the F0 alteration mid-vocalization (the “off” condition). The researchers hypothesized that if the F0 target was fixed, the speakers would compensate for the F0 alteration in the on condition, but not the off condition. Since the auditory feedback manipulation was imposed prior to vocalization onset in the off condition, the researchers reasoned that the speakers would not classify the auditory feedback as self-produced. Thus, if the speakers were comparing their auditory feedback to a fixed target, this comparison would not take place in the off condition, as the auditory feedback would not be perceived as the speaker’s own. Alternatively, if the speakers were using variable references, compensatory responses were expected in both conditions, as both the on and off manipulations created a sudden change in the F0 of the speaker’s auditory feedback. The results of this study indicated that the size and timing of the compensatory responses were equivalent across the on and off conditions, providing support for the use of a variable F0 target when sustaining ongoing vocalizations.

Similarly, Hawco and Jones (2009) exposed speakers to FAF by altering the F0 of the speaker’s vocalizations at vocalization onset and then perturbing the vocalizations by briefly removing the F0 alteration. Speakers were also exposed to unaltered auditory feedback, which was then perturbed mid-utterance by introducing a brief F0 change. This design allowed the researchers to compare the responses to perturbations that introduced, as well as removed a F0 alteration. This design also allowed researchers to compare the responses to feedback alterations imposed at vocalization onset, relative to unaltered auditory feedback at vocalization onset. Much like the results reported by Larson et al. (2001), responses to the mid-utterance perturbations were identical regardless of whether the perturbation was introducing or removing a feedback alteration. However, responses to the feedback alteration imposed at vocalization onset were found to be much larger than any of the responses to mid-utterance perturbations. The researchers suggested the differences in responses to changes in F0 at vocalization onset and mid-utterance were the result of different control strategies used at these different time points. At vocalization onset, auditory feedback is compared to a sensory prediction generated by the motor system to ensure the correct F0 is produced (a fixed reference), while mid-utterance auditory feedback is compared with the most recently experienced F0 to stabilize the vocalization (a variable reference).

While recording vocal responses to FAF provides valuable information about the use of auditory feedback for speech motor control, neurophysiological measures, such as electroencephalography (EEG), provide information about the neural correlates of speech motor control. For example, Heinks-Maldonado et al. (2005) utilized EEG to demonstrate that auditory feedback resulting from the production of speech, is processed differently than sensory information generated by an external source. In this study, speakers were asked to produce vowel sounds while they listened to real-time playback of their unaltered voice, a pitch-shifted version of their voice, or an alien voice substituted for their own voice. Speakers also participated in a listening phase, where they heard recordings of their unaltered voice, a pitch-shifted version of their voice, and an alien voice played back to them. Examination of the auditory N1 event-related potential (ERP) revealed that speakers’ perception of their own unaltered voice resulted in a dampened sensory experience, or smaller N1 amplitudes, relative to the N1s elicited by the playback of their own unaltered voice, as well as the pitch-shifted and alien voice substituted versions of their voice in both the production and playback conditions. These results suggest that the neural processing of auditory feedback resulting from self-produced speech is suppressed (Heinks-Maldonado et al., 2005). Furthermore, the fact that this suppression was specific to the perception of self-produced unaltered speech, and not speech that was being altered in real-time, suggests that this suppression is not the result of a general dampening of all incoming auditory information during speech production, but rather a highly specific mechanism for processing the auditory consequences of self-produced speech (Heinks-Maldonado et al., 2005, 2006; Chen et al., 2012a).

In the present study, we utilized ERP responses to further investigate whether auditory feedback is compared to a fixed or variable reference during speech production. Vocal and ERP responses following FAF perturbations that either introduced a brief change to the F0 of the speaker’s auditory feedback, or briefly restored the F0 of a speaker’s auditory feedback to its unaltered state, were compared. If as suggested by Larson et al. (2001) and Hawco and Jones (2009), auditory feedback is compared to a variable reference after the vocalization is initiated, we would expect similar vocal and ERP responses across all perturbation conditions. However, if auditory feedback is compared to a fixed reference, we would expect the smallest vocal and ERP responses when the FAF perturbations cause the speaker’s auditory feedback to return to its unaltered state, relatively larger vocal and ERP responses when the speaker’s unaltered auditory feedback is perturbed, and the largest vocal and ERP responses when the speaker’s already altered auditory feedback is perturbed even more, reflecting the relative sizes of the mismatch between the perceived auditory feedback and the fixed reference.

## Materials and Methods

### Participants

Thirty-six participants between the ages of 18 and 23 years (mean 19.09 years; standard deviation (SD) 0.99 years; 27 females) participated in this study. Participants were right-handed native Canadian English speakers, who did not speak a tonal language. Participants completed a music experience questionnaire, and two participants reported having received formal vocal training. Participants gave informed consent and received course credit or financial compensation for their participation. This study was carried out in accordance with the recommendations of Wilfrid Laurier University Research Ethics Board. The protocol was approved by the Research Ethics Board. All subjects gave written informed consent in accordance with the Declaration of Helsinki.

### Procedure

**FIGURE 1:**
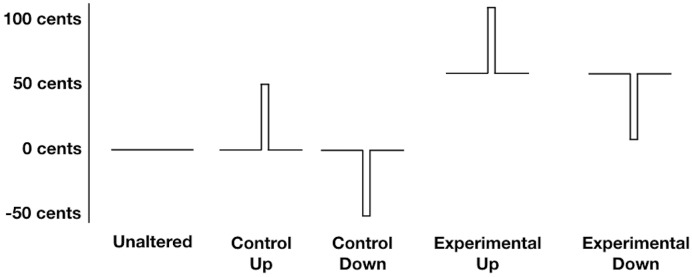
Schematic Representation of the five experimental conditions. During the unaltered condition participants listened to their unaltered auditory feedback. During the control up and control down conditions, the fundamental frequency of participants’ auditory feedback was unaltered at vocalization onset, but was then briefly perturbed upwards 50 cents, or downwards 50 cents, respectively. During the experimental up and experimental down conditions, the fundamental frequency of participants’ auditory feedback was raised by 50 cents at vocalization onset, and was then briefly perturbed upwards 50 cents (for a total deviation from baseline of 100 cents), or perturbed downwards 50 cents (returning participants fundamental frequency to baseline), respectively.

Participants vocalized the vowel sound /a/, 250 times over five blocks, while being exposed to unaltered feedback and FAF. Participants were cued to start vocalizing by a green box on a computer screen, while a red box indicated they should stop vocalizing. Each experimental block contained 50 trials in which the participants’ auditory feedback was briefly perturbed 50 cents (equivalent to half a semitone) upwards or downward, or left unaltered. Each perturbation had a fixed duration of 200 ms and occurred randomly between 1000 and 1800 ms after the onset of the vocalization. In addition, half of the perturbed trials were also manipulated so that at vocalization onset, and throughout the vocalization, the fundamental frequency of participants’ auditory feedback was 50 cents higher than their baseline fundamental frequency. Combined, these experimental manipulations resulted in five unique conditions, which were each randomly repeated 50 times across the five blocks for a total of 250 trials (see Figure 1). During the *unaltered condition* participants listened to their unaltered auditory feedback. During the *control up* and *control down* conditions, the fundamental frequency of participants’ auditory feedback was unaltered at vocalization onset, but was then briefly perturbed upwards 50 cents, or downwards 50 cents, respectively. During the *experimental up* and *experimental down* conditions, the fundamental frequency of participants’ auditory feedback was raised by 50 cents at vocalization onset, and was then briefly perturbed upwards 50 cents (for a total deviation from baseline of 100 cents), or perturbed downwards 50 cents (returning participants fundamental frequency to baseline), respectively.

### Apparatus

Participants wore a 32 channel NeuroScan Quik-Cap (Compumedics, Charlotte, SC, United States), Etymotic ER-3 insert headphones (Etymotic Research, Elk Grove Village, IL, United States), and a Apex575 condenser headset microphone (Apex Electronics, Pickering, ON, Canada), and were tested in an electrically shielded booth (Raymond EMC, Ottawa, ON, Canada). The onset and offset of each pitch perturbation, as well as the vocalization onset pitch manipulation, were controlled by midi commands sent by MAX/MSP 4 (Cycling ‘74, San Francisco, CA, United States). The midi commands triggered TTLs to be sent from custom made hardware to the Neuroscan system and the voice recording system (HD-P2; TASCAM, Montebello, CA, United States) to mark the onset and offset of the pitch perturbations, respectively. Voice signals were sent to a mixer (Mackie Onyx, 1220; Loud Technologies, Woodinville, WA, United States), followed by a digital signal processor (VoiceOne; T.C. Helicon, Victoria, BC, Canada), which altered the fundamental frequency of the voice signal. The altered voice signal was then presented back to the participant through headphones as FAF. The unaltered voice signal was digitally recorded at a sampling rate of 44.1 kHz for later analysis.

### Behavioral Recording and Analysis

The unaltered voice signal was segmented into separate vocalizations, and F0 values were calculated for each vocalization with the SWIPE’ algorithm (Camacho and Harris, 2008). Each vocalization was then segmented on the basis of the onset of the perturbation. Fundamental frequency values for each segment were then normalized to the baseline period, which was the portion of the segment 100 ms prior to the onset of the perturbation, by converting hertz values to cents with the formula cents = 100(12 log2 F/B), where F is the fundamental frequency value in hertz, and B is the mean fundamental frequency of the baseline period.

Cents values were calculated every millisecond for the 200 ms prior to the perturbation and the 500 ms after the perturbation. An averaged F0 trace was constructed for each experimental condition, for each participant.

Vocal responses were quantified by examining the response magnitude and latency. The magnitude of the compensatory response was determined by finding the point at which the participant’s averaged F0 trace deviated maximally from the baseline mean, between the window of 60–500 ms after perturbation onset. The latency was calculated as the time at which this maximal deviation occurred.

### ERP Recording and Analysis

EEG signals were recorded from 32 scalp electrodes and referenced online to electrodes placed on each mastoid. Data were bandpass filtered (1–30 Hz) and digitized at 1000 Hz. Electrode impedances were maintained below 5 kΩ throughout the duration of the experiment. After data acquisition, EEG voltage values were re-referenced to the average voltage across all electrode sites. The data were then epoched into segments from 100 ms before the perturbation onset to 500 ms after the perturbation onset. Data were analyzed offline for movement artifacts, and any segment with voltage values exceeding 55 μV of the moving average over an 80-ms span were rejected. In addition, a visual inspection of all the data was completed to ensure that artifacts were being adequately detected. 10 subjects were eliminated from further analyses, as they had less than 30 artifact-free trials per experimental condition. On average the included participants had 41 artifact-free trials per experimental condition.

Nine electrodes were included in the analysis: F3, Fz, F4, FC3, FCz, FC4, C3, Cz, and C4. These electrodes were then grouped into three electrode sites: left (F3, FC3, and C3), medial (Fz, FCz, and Cz), and right (F4, FC4, and C4). These electrodes were chosen on the basis of visual inspection of the regions showing the most robust N1-P2 components, as well as previous research suggesting that fronto-medial and centro-frontal regions are optimal for recording N1-P2 responses to FAF (Behroozmand et al., 2009; Chen et al., 2012b; Korzyukov et al., 2012; Scheerer et al., 2013a,b; Scheerer and Jones, 2014; Scheerer, 2016; Zhu et al., 2016).

For each participant, averaged waveforms were created for the unaltered and FAF conditions for each electrode. Grand-averaged waveforms were created for each of the five conditions by averaging the data from all participants for each electrode, and this was followed by baseline correction. For all averaged files for each participant, the peak amplitude and latency of the peak amplitude were calculated for the ERP components of the N1-P2 complex. On the basis of visual inspection of the latency of the most prominent ERP peaks in the grand-averaged ERP waveform, the components were extracted at time windows from 145–215 ms and 215–315 ms, respectively.

### Statistical Analyses

In order to investigate vocal response magnitudes and latencies, SPSS (v. 19.0) was used to conduct repeated measures analysis of variances (RM-ANOVAs) with experimental condition (unaltered, control up, control down, experimental up, and experimental down) as a within-subjects factor. Similarly, to investigate ERP response magnitudes and latencies, RM-ANOVAs were conducted with experimental condition and electrode site (left, medial, right) as within-subjects factors. Note that the unaltered condition was not used in the analyses of response latencies, as stimuli were not presented during the unaltered trials, thus data were randomly sampled with no true reference, rendering latency information meaningless. It is also important to note that while pooling across up and down conditions would increase the total number of experimental and control trials in our comparisons, these conditions are not equal, particularly in the experimental conditions. In the experimental up condition, perturbing the already altered vocalization + 50 cents resulted in a cumulative + 100 cent manipulation. While in the experimental down condition, perturbing the already altered auditory feedback -50 cents essentially negated the alteration at vocalization onset. Since we cannot assume that the brain processes these perturbations in isolation (and does not consider the cumulative error size), we analyzed the upward and downward perturbations separately. Significant main effects were followed up with bonferroni corrected *t*-tests, while significant interactions were followed up with one-way ANOVAs and bonferroni corrected *t*-tests. The Greenhouse-Geisser (Greenhouse and Geisser, 1959) correction was used in instances where Mauchley’s assumption of sphericity was violated. However, original degrees of freedom were reported for ease of interpretation.

## Results

### Vocal Response Magnitude

**FIGURE 2:**
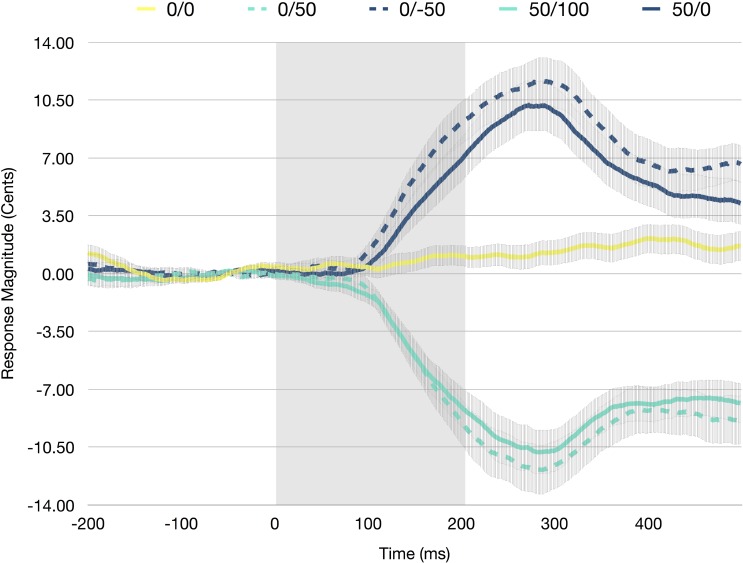
Averaged F0 trace averaged across all participants for the unaltered (0/0), control up (0/50), control down (0/-50), experimental up (50/100), and experimental down (50/0) conditions. Error bars represent the standard error of the mean. The shaded portion (0–200 ms) indicates the perturbed portion of the trace.

A RM-ANOVA was conducted to investigate the influence of experimental condition on vocal response magnitudes. There was a significant main effect of condition [*F*(4,100) = 118.018, *p* < 0.001, η^2^ = 0.825], as vocal responses in the unaltered condition were significantly smaller than both downward shifted conditions (*p* < 0.001) and significantly larger than both upward shifted conditions (*p* < 0.001). In addition, both the upward shifted conditions resulted in significantly smaller (more negative) vocal responses, than both the downward shifted conditions (*p* < 0.001; see Figure 2).

### Vocal Response Latency

A RM-ANOVA was conducted to investigate the influence of experimental condition on vocal response latency. Latencies were not found to differ significantly across the four conditions [*F*(3,75) = 0.910, *p* = 0.422, η^2^ = 0.035].

### ERP Responses

#### N1 Amplitudes and Latencies

**FIGURE 3:**
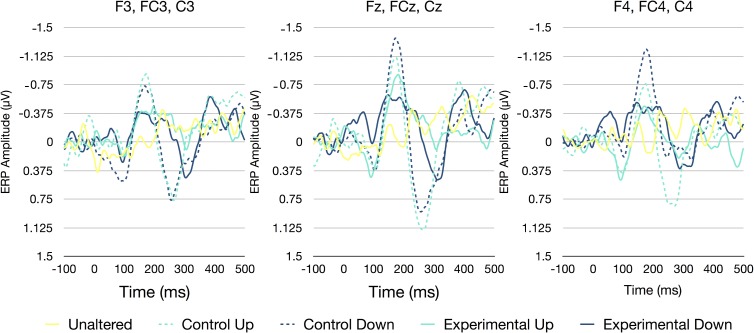
ERP waveforms averaged across all participants and all electrodes [left (F3, FC3, and C3), medial (Fz, FCz, and Cz), and right (F4, FC4, and C4)]. Separate lines represent each of the five perturbation categories unaltered (0/0), control up (0/50), control down (0/-50), experimental up (50/100), and experimental down (50/0).

A two-way RM-ANOVA was conducted to investigate the influence of experimental condition and electrode site on N1 amplitudes. There was a significant main effect of condition [*F*(4,100) = 8.742, *p* < 0.001, η^2^ = 0.259] and electrode site [*F*(2,50) = 3.907, *p* = 0.027, η^2^ = 0.135]. However, the interaction between condition and site was non-significant [*F*(8,200) = 1.413, *p* = 0.193, η^2^ = 0.054]. Bonferroni corrected *t*-tests indicated that the main effect of experimental condition was driven by significant differences between the control down condition and the experimental up (*p* = 0.004), experimental down (*p* = 0.010), and the unaltered (*p* < 0.001) conditions. The differences between the control up condition and the unaltered condition were also marginally significant (*p* = 0.068). With regards to electrode site, the N1 amplitudes at the medial sites and were marginally larger than those at the left lateralized sites (*p* = 0.068; see Figure 3).

A two-way RM-ANOVA was also conducted to investigate the influence of experimental condition and electrode site on N1 latency. The main effect of condition [*F*(3,75) = 1.419, *p* = 0.244, η^2^ = 0.054], electrode site [*F*(2,50) = 0.717, *p* = 0.457, η^2^ = 0.028], and the interaction between condition and electrode site [*F*(6,150) = 0.775, *p* = 0.591, η^2^ = 0.030], were all non-significant.

### P2 Amplitudes and Latencies

A two-way RM-ANOVA was conducted to investigate the influence of experimental condition and electrode site on P2 amplitudes. There was a significant main effect of condition [*F*(4,100) = 12.270, *p* < 0.001, η^2^ = 0.329], electrode site [*F*(2,50) = 4.480, *p* = 0.016, η^2^ = 0.152], as well as an experimental condition by electrode site interaction [*F*(8,200) = 3.816, *p* = 0.003, η^2^ = 0.132]. Bonferroni corrected *t*-tests indicated that the main effect of experimental condition was driven by significant differences between the control up condition and both the experimental up (*p* < 0.001) and the unaltered (*p* = 0.001) conditions. Similarly, the control down condition was also significantly different than the experimental up (*p* < 0.001) and the unaltered condition (*p* = 0.001), and marginally different than the experimental down condition (*p* = 0.070). With regards to electrode site, the P2 amplitudes at the medial sites were larger than those at the right lateralized sites (*p* = 0.045), while P2 amplitudes at the medial sites were marginally larger than those at the left lateralized sites (*p* = 0.097). The interaction between experimental condition and electrode site appeared to be driven by large differences between the control conditions and both the experimental and unaltered condition at the left and medial electrode sites, while only the control up condition appeared to be different than the other experimental conditions at the right lateralized sites (see Figure 3).

A two-way RM-ANOVA was also conducted to investigate the influence of experimental condition and electrode site on P2 latency. The main effect of condition was significant [*F*(3,75) = 5.846, *p* = 0.001, η^2^ = 0.190], as responses peaked faster in both the control up condition, relative to the experimental up (*p* = 0.004) and the experimental down (*p* = 0.001) conditions. However, the main effect of electrode site [*F*(2,50) = 0.097, *p* = 0.871, η^2^ = 0.004] and the interaction between condition and electrode site [*F*(6,150) = 0.806, *p* = 0.567, η^2^ = 0.031], were non-significant.

## Discussion

The aim of this study was to use both vocal and ERP responses to probe whether auditory feedback is compared to a variable or fixed reference during ongoing speech. To that end, vocal and ERP responses were recorded following F0 perturbations to speakers’ unaltered auditory feedback, and also when F0 perturbations were imposed by briefly removing, or further increasing, the F0 manipulation that was introduced prior to vocalization onset. Similar to the results reported by Larson et al. (2001) and Hawco and Jones (2009), compensatory vocal responses were similar regardless of whether FAF perturbations were induced by briefly introducing, removing, or further increasing a F0 manipulation. These results suggest that auditory feedback is compared to a variable reference during ongoing speech. However, upon examination of the ERP responses, only N1 and P2 amplitudes elicited by auditory feedback perturbations occurring to the speakers’ unaltered auditory feedback, not their altered feedback, were found to be larger than the ERPs recorded during the unaltered auditory feedback condition (no perturbation control condition). In light of the pattern of the obtained vocal responses, the fact that perturbations to unaltered auditory feedback, but not altered auditory feedback, elicited large N1 and P2 responses suggesting that the magnitude of these ERP responses not only reflects a comparison between the current auditory feedback and the auditory feedback perceived prior to the FAF manipulation, but also reflects activity in the motor system as a result of the comparison of ongoing auditory feedback to the sensory prediction created by the motor system.

When speech motor commands are executed, the motor system also sends a copy of the expected sensory consequences, or a sensory prediction, of those motor commands to the auditory cortex (Guenther et al., 2006; Hickok and Poeppel, 2007; Houde and Nagarajan, 2011; Houde and Chang, 2015). When incoming sensory feedback matches the sensory prediction, a net cancelation occurs. This cancelation results in suppressed neural activation during the perception of self-generated sensory feedback, relative to externally generated sensory feedback (Weiskrantz et al., 1971; Blakemore et al., 1998, 2000; Heinks-Maldonado et al., 2005, 2006; Sitek et al., 2013). Specifically, Heinks-Maldonado et al. (2005, 2006) found that auditory cortical responses were suppressed while speakers listened to their unaltered auditory feedback during active vocalization, relative to when they listened to auditory feedback that was being altered in real time. Based on these findings, we propose that in the control conditions, activity in auditory cortical regions was suppressed as the participant’s auditory feedback matched the sensory prediction created by their motor system. It was not until the perturbation occurred that there was a mismatch between the expected and actual auditory feedback. As a result of this mismatch, activity in the auditory cortical regions increased, which was observed as large N1 and P2 responses. On the other hand, in the experimental conditions, since auditory feedback was in violation of the prediction created by the motor system immediately after vocalization onset, suppression did not occur. Since these auditory cortical areas were already active due to this violation of the sensory prediction, the response to the mid-vocalization perturbation was masked by the higher baseline activity in these regions. As a result, relatively smaller N1 and P2 responses were observed in the experimental conditions, relative to the control conditions.

An alternative possibility is that the obtained pattern of results reflects the relative weighting of feedback and feedforward input across the different vocalization conditions. Models of speech motor control, such as the directions into the velocity of articulators model (DIVA), describe fluent speech production as being the byproduct of the coordinated efforts of a feedback and feedforward control system (Houde and Nagarajan, 2011; Tourville and Guenther, 2011; Guenther and Vladusich, 2012; Kim and Max, 2014; Kittilstved et al., 2018). The feedforward control system operates by activating sensorimotor representations associated with a target sound (Tourville and Guenther, 2011). Once the sensorimotor representation has been activated, both the feedback and feedforward systems send motor commands to the primary motor cortex (Tourville and Guenther, 2011; Guenther and Vladusich, 2012). The motor commands executed by the feedforward system travel from the sensorimotor representation to articulatory control units in the cerebellum, before arriving at the primary motor cortex. Whereas, the motor commands executed by the feedback system pass through auditory and somatosensory feedback control subsystems, before reaching the primary motor cortex. As both the feedback and feedforward systems provide input to the premotor cortex, the overall motor command reflects the combined effort of these two systems (Tourville and Guenther, 2011; Guenther and Vladusich, 2012). Feedback control is particularly important during development as it allows the associations between particular motor movements and their sensory consequences to be learned. Overtime as development halts and the relationship between speech motor movements and their sensory consequences become more stable, the feedforward system becomes capable of producing the intended speech target without error. As a result, feedback input becomes relatively less important for fluent speech production, unless unexpected sensory feedback is perceived (Civier et al., 2010; Tourville and Guenther, 2011). That being said, recent research suggests that the relative weighting of feedback versus feedforward control can be context dependent. For example, Scheerer and Jones (2014) demonstrated that exposing participants to deviant auditory feedback that was predictable in magnitude resulted in smaller vocal and N1 ERP amplitudes relative to auditory feedback perturbations that were unpredictable in magnitude. It was suggested that when the magnitude of the auditory feedback perturbations was predictable, the perturbations were more readily distinguished from self-produced variability and deemed externally produced. After continuous exposure to these predictable perturbations, weighting of the feedforward system increased. This is because auditory feedback is only useful for regulating speech production when it is providing information relevant to controlling the articulators in such a way that it will allow for the intended sound to be produced. When auditory feedback does not reflect the current state of the articulators, the information is no longer useful for producing the intended sound, thus feedforward control is increased. When feedforward control is increased, auditory feedback is less salient, thus vocal and ERP responses to deviant auditory feedback are smaller. We suggest that a similar phenomenon may be occurring here, where in the experimental condition exposure to deviant auditory feedback at vocalization onset led to an increased weighting of feedforward input, resulting in the mid-vocalization auditory feedback perturbations being less salient. The decreased salience of these mid-vocalization perturbations led to the smaller N1 and P2 responses observed here. Although this explanation would also predict smaller vocal responses, which statistically were not observed, qualitatively vocal responses were smaller in the experimental condition.

The results of the current study suggest that ERP responses to mid-utterance perturbations may reflect not only the comparison of the current auditory feedback with the most recently experienced F0 to stabilize the vocalization (a variable reference), but also a comparison of the current auditory feedback to the sensory prediction generated by the motor system. However, it is unclear whether the ERP differences observed across the control and experimental conditions are the result of decreased neural suppression induced by the deviant auditory feedback at vocalization onset masking responses to the mid-vocalization feedback perturbations, or differences in the weighting of feedback and feedforward control across these two conditions. By exploring responses not only to the mid-utterance perturbations, but also at vocalization onset, further insight into how neural suppression at vocalization onset may modulate responses to subsequent perturbations may be obtained. Further exploration of the plausibility of the two potential explanations posed here will require future experiments with a larger number of trials than used for this study to achieve a strong enough signal to noise ratio to address these important questions.

## Author Contributions

NS and JJ conceived and designed the experiments and wrote the manuscript. NS performed the experiments and analyzed the data.

## Conflict of Interest Statement

The authors declare that the research was conducted in the absence of any commercial or financial relationships that could be construed as a potential conflict of interest.
